# Electro-Optical Properties of Sputtered Calcium Lead Titanate Thin Films for Pyroelectric Detection

**DOI:** 10.3390/mi11121073

**Published:** 2020-12-01

**Authors:** Elham Mafi, Nicholas Calvano, Jessica Patel, Md. Sherajul Islam, Md. Sakib Hasan Khan, Mukti Rana

**Affiliations:** 1 Division of Physics, Engineering, Mathematics and Computer Sciences, and Optical Science Center for Applied Research, Delaware State University, Dover, DE 19901, USA; emafi@desu.edu (E.M.); npcalvano11@students.desu.edu (N.C.); jbpatel14@students.desu.edu (J.P.); 2 Department of Electrical and Electronic Engineering, Khulna University of Engineering and Technology, Khulna 9203, Bangladesh; sheraj_kuet@eee.kuet.ac.bd (M.S.I.); sakib@eee.kuet.ac.bd (M.S.H.K.)

**Keywords:** pyroelectric, thermal detector, infrared detector, polarization, lead titanate, PCT, uncooled detector

## Abstract

We report the deposition and characterization of calcium lead titanate (PCT) thin films for pyroelectric detectors. PCT films of thicknesses ranging from ~250 to 400 nm were deposited on both silicon and Si/SiN/Ti/Au substrates at 13 mTorr pressure by 200W radio frequency sputtering in an Ar + O_2_ environment. Substrates were kept at variable temperatures during the deposition. The PCT films were annealed at various temperatures in an O_2_ environment for 15 min. X-ray diffraction results confirm the polycrystalline nature of these films. Energy dispersive spectroscopy function of scanning electron microscope showed that the films are stoichiometric (Ca_0.43_Pb_0.57_) TiO_3_ (Ca/Ti = 0.5, Pb/Ti = 0.66). Temperature dependence of capacitance, pyroelectric current, and pyroelectric coefficient was investigated for different PCT films. Our results show that films deposited at 550 °C and 600 °C demonstrate better quality and larger values of the pyroelectric coefficient. On the other hand, the capacitance fabricated on the PCT films at 550 °C showed the highest value of pyroelectric current and pyroelectric coefficient which were 14 pA and at 30 °C was ~2 µC/m^2^K respectively at a higher temperature. In addition, we used density functional theory to determine the atomic and band structure, real and imaginary parts of dielectric constant and refractive index, and absorption and reflection constants with energy.

## 1. Introduction

Uncooled infrared detectors are one of the opto-electrical sensors which are utilized in night vision cameras and scientific instruments such as radiometers and spectrometers. They have greater demand because of the capability to provide high-quality images with reduced cost, weight, and size. They also have lower power consumption, compared to the cool infrared detectors. Due to the advancement in lithography systems such as e-beam lithography and screen printing, it is possible to fabricate high-performance uncooled infrared detectors with smaller feature sizes.

Pyroelectricity is a temperature-dependent spontaneous polarization that creates permanent dipoles in the crystal. The system behaves as a pyroelectric material until it reaches a temperature known as its Curie point, beyond which the spontaneous change in polarization along with the change in temperature does not become affected. Pyroelectric materials have a relatively high dielectric constant and show spontaneous electric polarization without the presence of an applied electric field or stress. As a result, pyroelectricity arises from a polar material as the temperature along with a constant electric field and stress changes.

Pyroelectric detector’s temperature increases when the absorption of incident radiation from objects and their polarization changes. This change in polarization will ultimately vary onto the overall surface charge based on the temperature of the system. This electric charge is either attracted or repelled by the changing dipole. We can model these pyroelectric detectors as capacitors filled with pyroelectric materials. The detector will then observe the spontaneous polarization change as the temperature of the system begins to change. A material with a larger pyroelectric coefficient will produce a larger pyroelectric current (*i_p_*) as indicated in Equation (1) [1].

(1) $$i_{p}=p\frac{dT}{dt}A_{d}$$

Here, *A_d_* is the area of the electrode, *p* is the pyroelectric coefficient, and *T* is the temperature. Therefore, if the temperature change is reversed (for instance, from heating to cooling), the current flow will also reverse. Thus, pyroelectricity is both a reversible effect and a dynamic effect—it only responds to changes in temperature. Due to these properties of pyroelectric materials, they are used as the sensing layer of thermal detectors. Pyroelectric detectors have shown significant importance over the course of their applications such as atmospheric temperature measurement, air quality monitoring, motion sensors, IR spectrometers, and fire alarms, etc. One of the many advantages of pyroelectric detectors is that they do not require any bias current to operate.

Some of the most commonly used pyroelectric materials are modified lead titanates such as lead oxide zirconium titanate (PZT), calcium modified lead titanate (PCT), and aluminum nitride (AlN) [2]. These materials have been shown to gain crystal growth from thin film deposition, especially by using the radio frequency (RF) magnetron sputtering technique. It is one of the physical vapor deposition (PVD) methods where the substrate acts as an anode and the target acts as a cathode in a chamber. After pumping down to low vacuum (~10^−6^ Torr), RF power is applied between the electrodes. An ion beam is bombarded on a target material, and the magnetic field created from a magnet is used to confine the plasma toward the target. This enhances the ionization efficiency due to the orthogonal electromagnetic field formed on the target’s surface which increases the energy and ion density and leads to an increased rate of deposition [3,4,5,6,7]. These materials have been used for pyroelectric detection as they demonstrate high pyroelectric coefficients. Perovskite crystal structure is useful for higher pyroelectric coefficients. Lead titanate (PbTiO_3_) is one of the perovskite-crystal structures. Perovskite crystal structures also tend to have a high Curie temperature and can be modified by applying dopants to allow the controlling of certain parameters [8]. Modified lead titanates are created by adding Ca with PbTiO_3_ and have a higher possibility of creating this perovskite structure, hence they are one of the popular pyroelectric materials.

Modified lead titanate exhibits high permittivity, large spontaneous polarization, high Curie temperature, pyroelectric current, electro-optic, and ferroelectric effects at room temperature and is used in many different applications. These applications are included in infrared detectors such as in fire alarms, gas analyzers, intrusion detectors, optothermal detectors, pollution detectors, and position sensors as well as solar cell studies and engine analysis; in imaging devices such as in forest fire detection, pyroelectric vidicon, biomedical imaging, and natural resource surveillance; and in other applications such as differential temperature detectors, microwave detectors, X-ray detectors, actuators, energy harvesting devices, and optical switches [9,10]. The value of the pyroelectric coefficient in PCT depends on the growth of a high-quality ferroelectric perovskite structure.

In this work, we report the preparation of PCT thin films and also determine some of the structural, electrical, and optical properties of PCT thin films by theoretical and experimental methods. We used the RF sputtering technique with varying oxygen concentrations in an argon environment to deposit the PCT thin films of 600–650 nm thickness on Si and Si/SiN/Ti/Au substrates. Silicon was chosen as the deposition substrate because we obtained better quality PCT films on this substrate than the ones which were deposited on glass and sapphires. Au films of 550 μm radius were used to form the electrodes of the capacitive devices made of PCT films. We annealed the films at various temperatures to form the crystallinity and to determine the crystal orientations of the PCT film. Then, we experimentally determined the atomic compositions, crystal structure and size, and pyroelectric properties of thin films. We also demonstrated the atomic and band structures and some of the other electrical and optical properties of the PCT thin films through theoretical calculations using density functional theory (DFT). This work will help to understand some of the electrical, optical, and atomic properties of RF magnetron sputtered PCT thin films to utilize them in many applications which include uncooled infrared detection among others.

## 2. Materials and Methods

### 2.1. Calcium Modified Lead Titanate (PCT) Thin Film Deposition and Device Fabrication

PCT films were deposited on both Si and Si/SiN/Ti/Au substrates at 12–13 mTorr pressure by 200 W RF sputtering in an Ar/O_2_ (100–104/0 standard cubic centimeter per minute, SCCM) environment for four hours (deposition rate 2.7 nm/minute). Argon, one of the inert gases, was used to bombard the target and eject the target materials’ ion which was later deposited on the substrate. The varying oxygen flow rate affects the amount of oxygen that bonds to the elements in the sputtered thin film. A ProLine-PVD75 model sputtering system from Kurt J. Lesker Company (Pittsburgh, PA, USA) was used for this purpose. The substrates were kept at 550 °C, 600 °C, and 650 °C during the deposition process as summarized in Table 1. The deposited PCT thin films were annealed at different temperatures for 15 min with ramping rates of 30 °C/min and 120 °C/min. Prior to sputtering, the sputtering chamber was evacuated to ~1 to 2 × 10^−7^ Torr base pressure to avoid contamination with other gases present in the air.

In order to measure the capacitance and pyroelectric properties of as-deposited thin films, Si wafers were sonicated with trichloroethylene, acetone, methanol, and isopropyl alcohol for 2 min followed by 1-min HF (2% solution) etching to remove the silicon oxide layer. After this step, PCT films were deposited on these samples.

Sandwich-structured device fabrication started after Si/SiN wafers were cleaned with trichloroethylene, acetone, methanol, and isopropyl alcohol for 2 min before Ti/Au deposition. Then, we deposited a 20 nm thick Ti layer, followed by a 100 nm thick Au layer. Ti layer served as the adhesion layer of Au to SiN and Au served as the bottom electrode and a seed layer for PCT thin films. Then, a 600–650 nm thick PCT layer was deposited.

**Figure 1:**
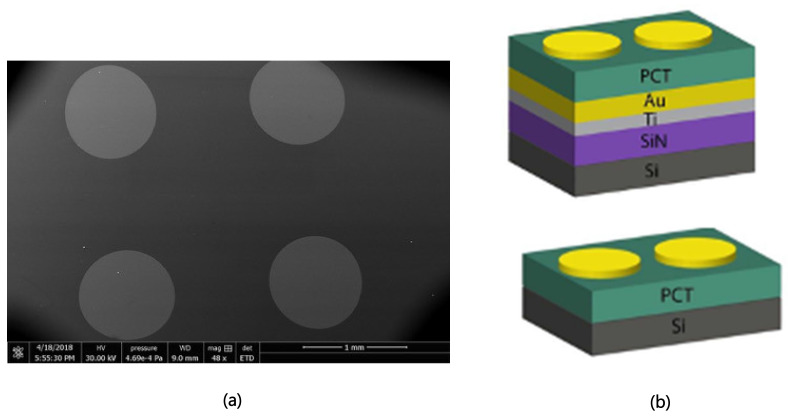
(**a**) Scanning electron micrograph of fabricated capacitors on top of the PCT film. (**b**) The cross-sectional view of the two types of capacitors tested.

After this, we deposited a 150 nm thick Au layer on both types of substrates. To pattern the Au for using it as an electrode for the capacitor of the PCT pyroelectric device, we used the liftoff technique. Negative photoresist NR9-3000PY was spin-coated at 6000 RPM for 45 s followed by a soft bake at 120 °C for one minute on the PCT layer. Then, the photoresist was exposed under the photomask aligner followed by post bake at 120 °C for one min. The wafer was developed in RD6 photoresist developer for 30 s. Then the wafer was rinsed in DI water for 30 s and blow-dried with an N_2_ gun. The liftoff was performed submerging the wafer in acetone for two hours and then removed and rinsed with DI water and blow-dried with N_2_. Scanning electron micrograph and cross-sectional area of the completed devices are shown in Figure 1a,b, respectively**.**

To anneal the thin films at different temperatures, a rapid thermal annealing system with 1000 SCCM O_2_ gas flow was used while the temperature varied from 550 °C to 700 °C with 50 °C increments. The annealing time was 15 min. Annealing was done before depositing the top Au electrode.

### 2.2. Structural and Atomic Characterization of PCT Thin Films

The film thickness was measured using the Dektak model XT profilometer. The roughness of the thin films was measured using an atomic force microscope (AFM) from Bruker. An FEI model Quanta 250 scanning electron microscope (SEM) was used to examine the surface morphology of the films.

To determine the elemental composition of PCT films, we deposited and annealed our samples at different temperatures. The energy dispersive spectroscopy (EDS) function of SEM was used to determine the atomic composition of thin films. EDS is a simple and non-destruction method that had been used to analyze the thin films both qualitatively and quantitively with an accuracy within the ±5% relative error envelope [11].

The crystal structure of the PCT films was determined by using a Bruker Advance 360 XRD machine which uses a monochromatic Cu k-α1 source with *λ* = 1.5406 Å at 40 kV voltage and 40 mA current. The specimens were scanned from 2*θ* = 20° to 2*θ* = 50°.

The Scherrer equation was used to estimate the crystal sizes for PCT and films which can be expressed by Equation (2):

(2) $$B\left(2\theta \right)=\frac{k\lambda }{L\cos\theta }$$

Here, *B* is the full width half maximum in radians, *θ* is the Bragg angle in degrees, *L* is the crystal size, *k* is the Scherrer constant for spherical crystals with cubic symmetry (=0.94), and *λ* is the wavelength of the X-ray source used during the XRD process (=1.54 Å) [12].

These samples were then annealed with two different temperature ramping rates 120 °C/min and 30 °C/min**.**

### 2.3. Electrical Properties of PCT Films

To determine the capacitance and pyroelectric current at various temperatures, we used the temperature ramping technique. We used the simplified version of the Lang–Steckel technique as pointed out by Fabel and Henisch and mentioned by [13,14]. The PCT samples with a metal electrode made of Au of 550 μm radius were placed inside an electro-mechanically shielded micromanipulator probe station. The center-to-center distance between the electrodes was 220 mm. The measurements were done in the dark, and the temperature of the stage inside the probe station was varied from 30 °C to 80 °C with 5 °C increments using the automatic temperature controlling function of the micromanipulator probe station. For both device types indicated in Figure 1b, the thermal stimulus was applied through the Si substrate as that surface was touching the probe station’s chuck whose temperature was varied. The electrical polarization because of the change in temperature in the PCT layer was measured by probing between two metal Au electrodes as indicated by the circular contact pads in Figure 1. In this case, the current flows from one electrode to another.

Pyroelectric voltage was measured using a parameter analyzer from Agilent, model BA1500, while the capacitance was measured at 1 kHz using a model E4980A inductance, capacitance, and resistance (LCR) meter from Agilent. The pyroelectric voltage and the capacitance were used to determine the pyroelectric coefficient. Then the pyroelectric current was determined by using Equation (1).

### 2.4. Theoretical Calculation of PCT Thin Films

To determine the electrical and optical properties of a PCT thin film from a theoretical perspective, we also performed the first-principles calculation in the framework of density functional theory (DFT) with a plane wave basis set as implemented in the CAmbridge Serial Total Energy Package (CASTEP) platform suite [15]. This helped us to understand the atomic and band structure as well as some of the optical properties of PCT. The details of the calculation parameters are illustrated in Table 2.

The optical properties of PCT thin films were calculated in the range of 0 eV to 10 eV photon energy so that the significance of the materials for the infrared (IR), visible, and ultraviolet (UV) spectrum can be visualized. The optical properties were acquired through a series of calculations starting from the complex dielectric function as indicated by Equation (3) [15].

(3) $$\varepsilon \left(\omega \right)=\;\varepsilon_{1}\left(\omega \right)+i\varepsilon_{2}\left(\omega \right)$$

The dielectric function is directly correlated with electronic band-structures. It includes inter-band transition energies with momentum matrix. The complex dielectric function for this case was calculated using the energy momentum of occupied and unoccupied states by using Equation (4) [15]

(4) $$\varepsilon_{2}=\frac{2e^{2}\pi }{\Omega \varepsilon_{0}}\;\underset{k,v,c}{\sum }\left|\langle ,\psi_{k}^{c}|\hat{u\;}\right.\times r\left|\psi_{k}^{v},\rangle \right|2\;\delta \left(E_{k}^{c}-E_{k}^{v}-E\right)\;$$

where *e* is the electronic charge,$\;\hat{u\;}$ represents the vector defining the polarization of incident filed, Ω refers to the polarization density, r denotes the spatial position, and $\psi_{k}^{c}$ and $\psi_{k}^{v}$ are the conduction band (CB) and valence band (VB) wave-function at *k*, respectively.$\;E_{k}^{c}$, $E_{k}^{v}$, and E represent the conduction band energy, valance band energy, and Fermi energy, respectively.

From the Kramers–Kronig relationship, the real part $\varepsilon_{1}\left(\omega \right)$ was achieved [16]. The other optical properties, namely refractive index *n*(*ω*), extinction coefficient *k*(*ω*), optical reflectivity *R*(*ω*), and absorption coefficient *α*(*ω*), were computed from the complex dielectric function *ε*_2_ (*ω*) as mentioned in Equations (5)–(8) [16].

(5) $$n\left(\omega \right)=\sqrt{\frac{\left|\varepsilon \left(\omega \right)\right|+\varepsilon_{1}\left(\omega \right)}{2}\;}\;$$

(6) $$k\left(\omega \right)=\sqrt{\frac{\left|\varepsilon \left(\omega \right)\right|-\varepsilon_{1}\left(\omega \right)}{2}\;}$$

(7) $$R\left(\omega \right)=\frac{{\left(n-1\right)}^{2}+k^{2}}{{\left(n+1\right)}^{2}+k^{2}}$$

(8) $$\alpha \left(\omega \right)=\frac{2k\omega }{c}$$

## 3. Results

### 3.1. Electronic and Atomic Structure of PCT Films

**Figure 2:**
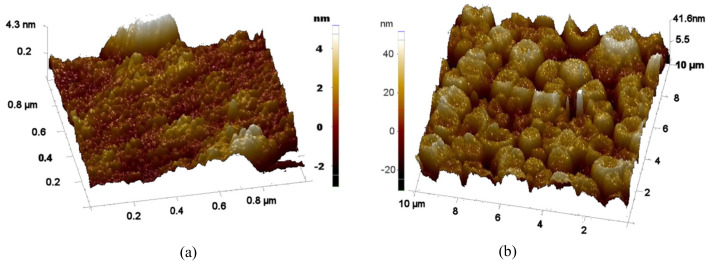
3D atomic force microscope (AFM) images of an as-deposited PCT film on Si substrate at 550 °C (**a**) without annealing and (**b**) after annealing at 600 °C for 15 min.

**Figure 3:**
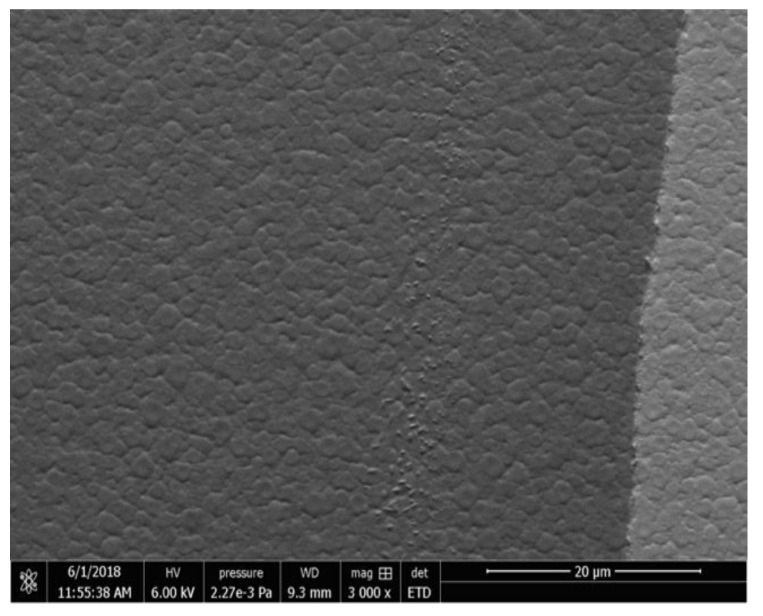
Scanning electron microscope (SEM) micrograph showing a crystal-growth PCT thin film annealed at 600 °C.

It was observed that the roughness of the film deposited at 550 °C without annealing is smaller (less than 8 nm) than the annealed one (more than 30 nm). Figure 2 shows this. The increase in surface roughness shows a visualization of the crystal growth during the annealing process. In other words, the form of crystals because of the annealing process increases the visible roughness of PCT films as shown in Figure 2b. The SEM micrographs were taken after the annealing of our films which revealed the morphology of crack-free PCT thin films which is shown in Figure 3. Under similar conditions, the deposition rate at room temperature was 27 Å/min, and the film thickness was measured ranging from ~600 to 650 nm.

EDS was used and the results reveal the presence of the elements such as calcium, lead, titanium, and oxygen in the PCT thin films. Moreover, they show that the films are stoichiometric whose atomic composition can be described as (Ca*_x_*Pb_1-*x*_) TiO_3_ where *x* = 0.41.

**Figure 4:**
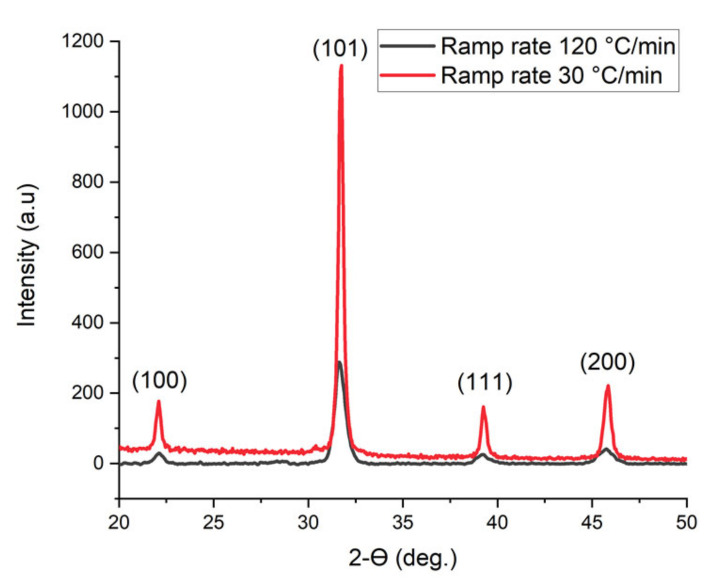
X-ray diffraction data for PCT thin films deposited at 550 °C and annealed at 550 °C for 15 min on silicon: temperature ramping rate 120 °C /min (black) and temperature ramping rate 30 °C/min (red).

Figure 4 shows the X-ray diffraction (XRD) data of PCT thin films both deposited and annealed at 550 °C with two different ramping rates −120 °C/min and 30 °C/min while annealing. It was observed that a lower ramping rate yielded sharper and taller peaks, indicating better quality and bigger crystal size for crystalline atoms of PCT. Calzadat et al. prepared PCT films by the soi-gel method and reported a formation of perovskite-structured Ca_0.24_Pb_o.76_TiO_3_ as well as monoclinic phase of PbTi_3_O_7_ [17]. They reported that the larger concentration of the PbTi_3_0_7_ phase was obtained in films crystallized with lower heating rates. They found that increasing the heating rate while annealing produces a diminution of the concentration of PbTi_3_O_7_. We observed a similar trend here where the higher heating rate was found to be detrimental to the crystallinity of the PCT films.

**Figure 5:**
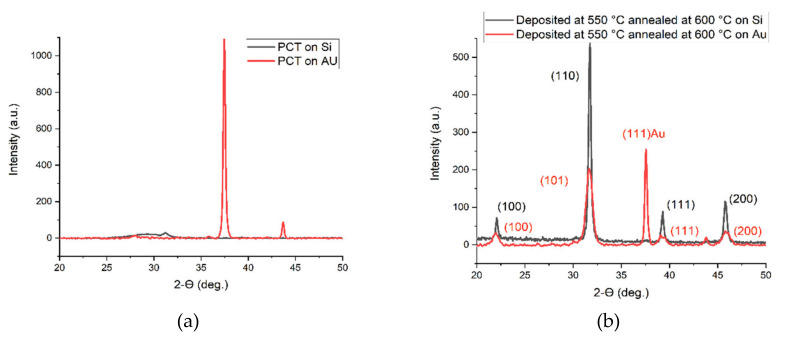
(**a**,**b**) show the XRD data of PCT thin films deposited on Si and Si/SiN/Ti/Au substrates before and after annealing, respectively.

A comparison was made between the crystal quality and the crystal sizes (for (101) orientation) of the films deposited and annealed at different temperatures on different substrates such as Si and Si/SiN/Ti/Au. It was observed that the substrate plays an important role on the quality and also crystal structure of the PCT films, their crystal sizes, and ultimately the pyro-coefficient. Figure 5a,b shows the XRD data of PCT thin films deposited on Si and Si/SiN/Ti/Au substrates before and after annealing, respectively.

It was found that as-deposited films (non-annealed) did not show any crystallinity (Figure 5a). This was also obvious from the AFM images shown in Figure 2, where the annealed samples shown in Figure 2b have a bigger crystal size as compared to the non-annealed case which is shown in Figure 2a. The peaks in red color in Figure 5a belong to the (111) direction of Au. It was observed that the structure of PCT film depends on the material on which it is deposited. For instance, films deposited on silicon have both tetragonal and cubic structures while films deposited on Au are mostly in the cubic or orthorhombic structures. As reported in the literature, the common crystalline structure for PCT seems to be perovskite tetragonal [18]. However, it is also reported that the amount of calcium in these films can play a crucial role in the formation of other crystalline structures. For example, it is reported that when the calcium content is higher than 40%, it can lead to the formation of cubic and orthorhombic phases [13]. For all the films, there were no obvious peaks that appeared between 25 °C and 30 °C of 2θ angles. This concludes that the PCT films studied for our case do not have pyrochlore structures generated from hexagonal PbO_1.37_ and orthorhombic α-PbO_2_ scrutinyite compounds [19].

It is seen in Figure 5b that films on silicon have sharper, stronger, and narrower peaks, indicating that silicon can be a better choice as a substrate as compared to Si/SiN/Ti/Au.

The XRD data on all of the annealed samples showed narrow and sharp peaks indicating high-quality crystallites that were developed in these samples. Samples deposited and annealed at different temperatures showed the highest peak at a two-theta angle of 32.45° and the second-highest peak at 46.55° 2θ angles. These peaks correspond to (101) and (200) crystal on the orientation of either tetragonal perovskite or cubic PCT film structures, respectively. We observed a peak at 22.8° for our annealed samples which corresponds to (100) crystal orientations of the PCT film. Thin films annealed at higher temperatures such as 700 °C also show polycrystalline structures with no pyroelectric current signal.

Table 3 shows the calculated average crystal size of annealed PCT films using Equation (2) for the strongest peaks (110) for tetragonal and (101) for cubic structures. The films deposited at 550 °C and annealed at 600 °C on Si substrate showed the largest crystal size among all, while the PCT films deposited on Si/SiN/Ti/Au substrate demonstrated the smallest crystal sizes. The crystal size plays an important role in the electrical properties of the thin films. Later, it was observed that the capacitance value for the PCT films on the Si wafers is two orders of magnitude larger than the ones on the Si/SiN/Ti/Au. It can be seen from Table 3 or Figure 4 and Figure 5 that the PCT films deposited on Si substrates have a larger crystal size or taller XRD peaks than those deposited on Si/SiN/Ti/Au substrate. This needs further investigation to explain it better.

The granular structure of the PCT thin film can also be seen from the SEM micrograph in Figure 3a. We also saw that a similar surface structure of other annealed samples, except the ones deposited and annealed at a higher temperature such as 700 °C, had obvious cracks.

**Figure 6:**
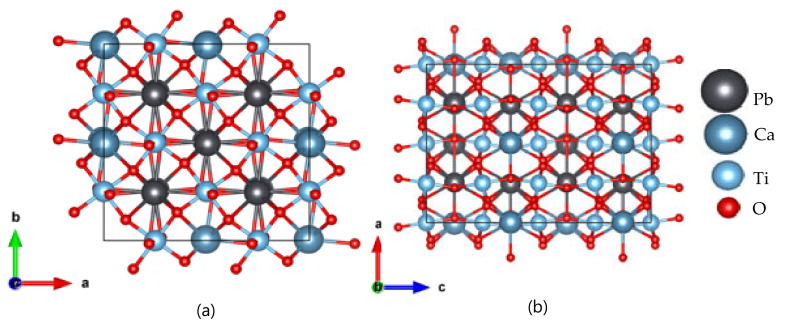
Optimized atomic structure of (Pb_0.593_Ca_0.406_) TiO_3_ films. (**a**) Side view; (**b**) Top view.

**Figure 7:**
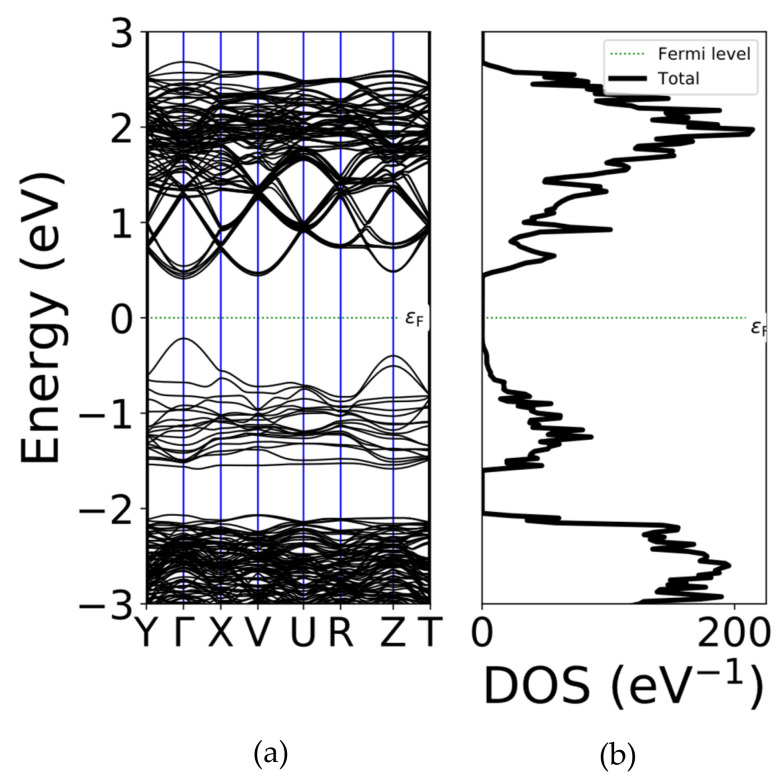
(**a**) Electronic band diagram and (**b**) density of states (DOS) for the (Pb_0.593_Ca_0.406_) TiO_3_ structure.

Figure 6a,b shows the optimized atomic structure of the PCT film—(Pb_0.593_Ca_0.406_)TiO_3_ determined by the DFT calculation. The triclinic lattice with optimized lattice parameters of a = 0.540 nm and c = 0.765 nm was obtained, which corresponds well with the crystallographic data [20]. The calculated electronic band structure and corresponding density of states (DOS) of (Pb_0.593_Ca_0.406_)TiO_3_ films are shown in Figure 7a,b, respectively. A direct bandgap of ~0.627 eV at the Γ point was observed for this structure. The electronic DOS further confirmed the bandgap of the film. This bandgap value is also in the infrared (IR) range, indicating the probable application of the material in an IR detector.

### 3.2. Electrical Properties of PCT Films

**Figure 8:**
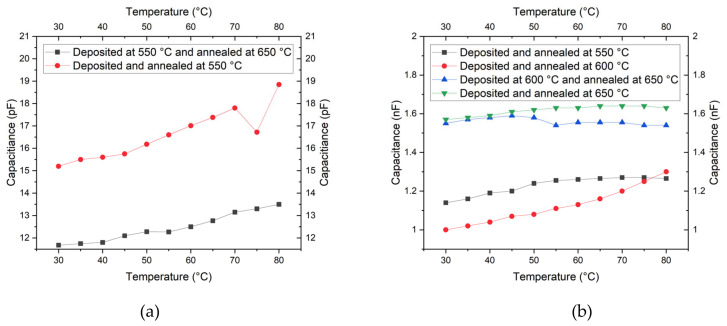
Variation of Capacitance for the PCT films deposited on (**a**) Si/SiN/Ti/Au and (**b**) Si substrates, respectively.

During this investigation, we saw that substrate plays a big role in the electrical properties of PCT thin films. Thin films on silicon substrate show a higher capacitance value (nF) compared to the ones on the Au (pF). The results are shown in Figure 8a,b for the PCT films deposited on Si/SiN/Ti/Au and Si substrates, respectively. It is also seen that the capacitance value slightly increases with the temperature variation. This increase in capacitance values by increasing the temperature suggests that a PCT-thin-film-based photodetector will have a higher sensitivity to temperature change. Films deposited on Si substrates had sharper and taller XRD peaks, and they mostly correspond to perovskite structures as compared to the one which was deposited on Si/SiN/Ti/Au substrates and has cubic structures [19]. The slight increase in capacitance with an increase in temperature as well as a sudden drop in the capacitance value of the PCT sample at 75 °C which was deposited and annealed at 550 °C is unknown.

**Figure 9:**
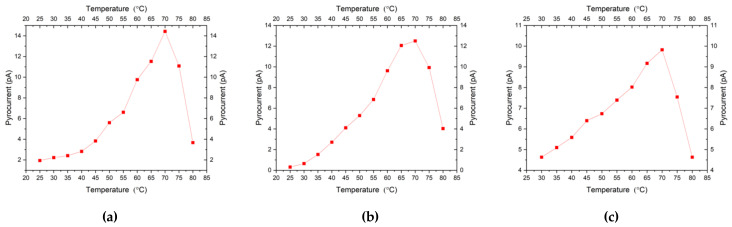
Variations of the pyroelectric current of PCT samples deposited and annealed, respectively, at (**a**) 550 °C–600 °C, (**b**) 550 °C–550 °C, and (**c**) 600 °C–600 °C.

Figure 9a–c describes the variations of the pyroelectric coefficient with temperature for the PCT thin films deposited on Si substrate. For all annealed PCT films, we observed that as the temperature increases, the pyroelectric currents and the pyroelectric coefficients also increase. The highest current value and the smoothest trend of increase in pyroelectric current were found for the PCT films deposited at 550 °C and annealed at 600 °C. For films deposited at 550 °C and annealed at 600 °C, the pyroelectric current through the capacitors starts at 2 pA at 30 °C and increases with temperature to 14 pA at 70 °C. Samples annealed at other temperatures showed a similar trend, although the PCT film deposited at either 550 °C or 600 °C shows better crystal qualities as well as a higher pyrocurrent. Our results of the increase in the pyroelectric current for PCT samples followed a similar trend reported in other studies [19,20,21,22,23,24,25,26,27,28].

**Figure 10:**
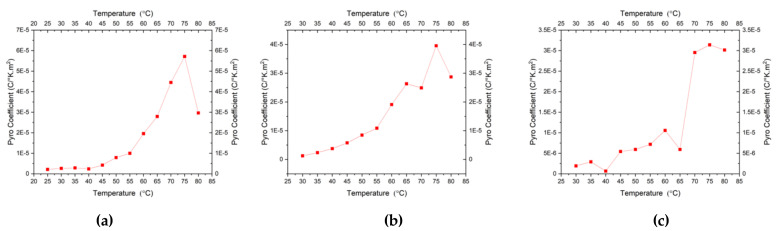
Variations of the Pyro-coefficient of PCT samples deposited and annealed, respectively, at (**a**) 550 °C–600 °C, (**b**) 550 °C–550 °C, and (**c**) 600 °C–600 °C.

The pyroelectric coefficients also increased with temperature as shown in Figure 10a–c. It can be seen that the best value obtained for the pyroelectric coefficient at room temperature (30 °C) was from the film deposited at 550 °C and annealed at 600 °C sample which is ~2 µC/(m^2^·K). This value is lower than the values reported by others [18,19,21]. Similar to the pyroelectric current, the 550 °C and 600 °C deposited and annealed PCT films had smoother trends among all. From Table 3, it can be seen that the film deposited at 550 °C and annealed at 600 °C has the largest crystal size as compared to the others which were deposited and annealed at different temperatures. The films deposited and annealed both at 550 °C and deposited and annealed both at 600 °C have a similar value of crystal sizes, although the former one demonstrated the pyroelectric coefficient and pyroelectric current one order of magnitude higher than the latter one. This needs more investigation to explain further. Table 4 shows the comparison of various materials, including one we investigated in this study. It can be seen from Table 4 that the pyroelectric coefficient of PCT films under current investigation shows similar values of the pyroelectric coefficient as reported by the other group.

### 3.3. Optical Properties of PCT Thin Films

**Figure 11:**
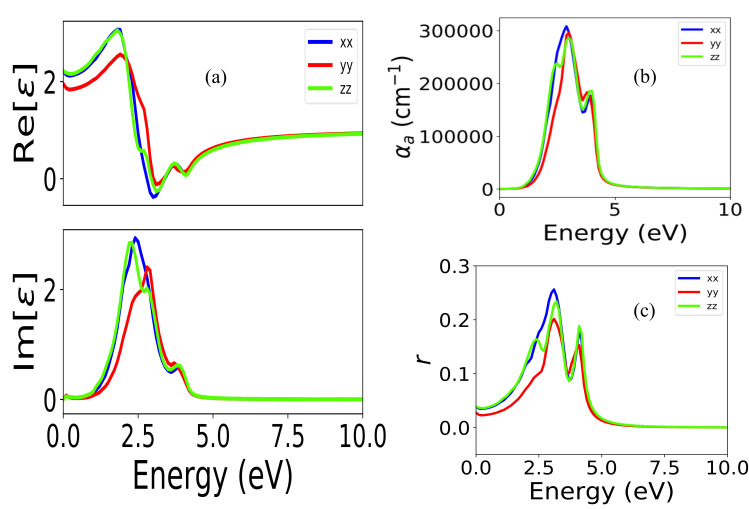
(**a**) Dielectric function, (**b**) absorption co-efficient, and (**c**) reflectivity as a function of energy for the (Pb_0.593_Ca_0.406_) TiO_3_ alloys.

Figure 11a shows the variation of real and imaginary parts of the complex dielectric function with energy. Here, xx, yy, and zz are the direction of the incident light. In the imaginary part of the dielectric function highest peak for x, y, and z incident lights are 2.43 (at 2.5 eV), 2.2 (at 3.1 eV), and 2.40 (at 1.89 eV).

Figure 11(a) Dielectric function, (b) absorption co-efficient, and (c) reflectivity as a function of energy for the (Pb_0.593_Ca_0.406_)TiO_3_ alloys.

Respectively. In the real part of the dielectric function, in the 3.1 eV to 3.4 eV photon energy range, the values become negative, representing semi-metallic nature at this energy region. Static dielectric is about 2.2 ε_0,_ 1.98 ε_0,_ and 2.2 ε_0_ for x, y, and z incident light, respectively. Static values also indicate strong anisotropy in the dielectric function in this material. Figure 11b shows the variation of reflectivity with energy between 0 to 10 eV. The highest reflectance is 28% at 2.75 eV for x incident light. In the UV region, there is another peak (19%) which is at 4.25 eV. The values become zero starting from 5.2 eV to 10 eV in the UV region. The first reflectance peaks for x, y, and z incident lights are 28% at 2.75 eV, 17% at 2.5 eV, and 19% at 2.76 eV, respectively. Static values are 4%, 3%, and 4% for x, y, and z incident lights, respectively. It shows the low reflectivity (around 4%) in the IR range of (0.01 eV to 1.1 eV). Figure 11c shows the variation of the absorption coefficient with energy between 0 to 10 eV. From the graph, it can be seen that the highest value of the absorption coefficient occurs at 2.78 eV, and the corresponding value is 3.1 × 10^5^ cm^−1^. The second peak of the absorption coefficient occurs in the UV region at 4.75 eV. The average optical bandgap was found to be 0.348 eV. First absorption peaks occur at 2.78 eV (3.1 × 10^5^ cm^−1^), 2.79 eV (2.9 × 10^5^ cm^−1^ ), and 2.5 eV (2.4 × 10^5^ cm^−1^) for x, y, and z incident lights, respectively. In the IR region, the absorption is around is 1 × 10^4^ to 1 × 10^5^ cm^−1^ cm^−1^. The absorption becomes zero at 5.4 eV. These findings suggest that low reflectance and high absorption in the infrared (IR) show the applicability of this material for IR detector technology and pyrolytic applications.

## 4. Conclusions

We deposited PCT in the Ar environment at room temperature on an Au electrode by RF magnetron sputtering, and the PCT thin films were annealed at 550, 600, and 650 °C in the presence of O_2_. The annealed PCT thin films showed polycrystallinty and both perovskite and cubic structures with strong and narrow peaks indicating the high quality of our films. The best pyroelectric coefficient at a higher temperature among all the samples was 50 µC/m^2^K which belongs to the PCT film deposited and annealed at 550 °C and 600 °C, respectively. To complement the experimental findings, we also determined the atomic structure as well as the electrical and optical properties of the PCT thin films by using DFT calculations. A direct bandgap with optically anisotropic characteristics was found from a typical (Pb_0.593_Ca_0.406_) TiO_3_ film. We need to investigate further to increase the pyroelectric coefficient for the films deposited on gold by improving the quality of these films. Increasing the substrate temperature, gas flow, and pressure during deposition and annealing temperature and time could be some of the variables that need to be optimized.

## Figures and Tables

**Table 1:** EDS results for calcium modified lead titanate (PCT) film deposited at 550 °C.

Deposition Temperature (°C)	Annealing Temperature (°C)	Composition	Pb/Ti	Ca/Ti	Ca	Pb	Ti	O
550	550	(Ca_0.41_Pb_0.59_) TiO_3.04_	0.70	0.50	8.9	12.5	17.8	54.2
550	600	(Ca_0.41_Pb_0.59_) TiO_2.80_	0.63	0.44	8.6	12.2	19.2	53.8

**Table 2:** Density functional theory **(**DFT) calculation parameters for ground state energy and geometry optimization.

Supercell	K-Mesh	Energy Cut-Off	Functional	Pseudo-Potential	Convergence Accuracy	Force Cutoff	Displacement Cutoff
**1 × 1 × 1**	3 × 3 × 3 5 × 5 × 5 For optical calculation	125.0 Hartree	GGA-PBE	Ultra-soft	$2\times 10^{-5}\;\mathrm{eV}$	$5\times 10^{-2}\;\mathrm{eV}/Å$	$2\times 10^{-3}\;Å$

**Table 3:** Crystal size estimation of deposited and annealed PCT films.

Sample	Deposition Substrate	Deposition Temperature (°C)	Annealing Temperature (°C)	Crystal Size (nm)
Sample#1	Si	550	550	28.9
Sample#2	Si	550	600	35.3
Sample#3	Si	600	600	28.99
Sample#4	Si	600	650	28.99
Sample#5	Si	650	650	29.28
Sample#6	Si/SiN/Ti/Au	550	600	11.5
Sample#7	Si/SiN/Ti/Au	550	650	11.5

**Table 4:** Comparison of the pyroelectric coefficients of various materials. RF: radio frequency.

Material	Thin Film Deposition Method	Pyroelectric Coefficient (µC/m^2^K)	Reference
PbZr_0.52_Ti_0.48_O_3_	Atomic layer deposition	380	[17]
AlN	Sputter	1.9	[18]
PVDF/PVC/BaTiO_3_ blend nanocomposites	Solution casting technique	351	[19]
(1-*x*) KNbO_3_-*x*BaNi_1/2_Nb_1/2_O_3-δ_ (KBNNO)	Solution-based	26	[20]
Hf_1-*x*_Zr*_x_*O_2_	Sputtering	48 for *x* = 0.64	[21]
(Pb_0.97_La_0.02_) (Zr*_x_*Sn_0.89−*x*_Ti_0.11_) O_3_ (*x* = 0.68)	Solution-based	680	[22]
Pb_0.7_Ca_0.3_TiO_3_	Pulse laser deposition	47	[15]
(Pb_0.593_Ca_0.406_) TiO_3_	RF sputter	2	Current Study
